# Genome editing and regeneration pipeline for engineering disease resistance in tomato using CRISPR/Cas9

**DOI:** 10.3389/fpls.2026.1754287

**Published:** 2026-03-24

**Authors:** İbrahim Erdoğan, Ruma Debbarma, Minu Sherry, İbrahim Mancak, Murray Grant, Mahmut Tör

**Affiliations:** 1 University of Warwick, School of Life Sciences, Elizabeth Creak Horticultural Technology Centre (ECHTC), Coventry, United Kingdom; 2 Manier Seeds, Adana, Türkiye; 3 Molecular Plant and Microbial Biosciences Research Unit, School of Science and the Environment, University of Worcester, Worcester, United Kingdom

**Keywords:** crop improvement, disease resistance, genome editing, susceptibility gene, targeted mutations

## Abstract

CRISPR/Cas9 (Clustered Regularly Interspaced Short Palindromic Repeats/Cas9)-based genome editing has emerged as a powerful tool for developing disease-resistant crops. Here, we present a comprehensive and reproducible protocol for applying CRISPR/Cas9 genome editing in tomato (*Solanum lycopersicum*), covering guide RNA (gRNA) design using CRISPOR, Golden Gate vector assembly, *Agrobacterium*-mediated transformation, plant regeneration, and molecular validation of edited plants. The workflow integrates standardized bioinformatics and sequencing-based validation tools, including DSDecodeM, TIDE, and protein-level impact analysis, to confirm targeted mutations and assess editing efficiency. Quantitative benchmarks for regeneration, transformation, and editing efficiencies were provided to support reproducibility. This protocol offers an integrated pipeline for generating and validating targeted gene knockouts in tomatoes and is intended to facilitate functional genomic studies and the development of disease-resistant cultivars. However, it is more widely applicable to gene editing in tomato plants.

## 1 Introduction

Tomato (*Solanum lycopersicum*) is one of the most important vegetable crops worldwide, contributing substantially to global vegetable production and human nutrition. It is a major dietary source of carotenoids, particularly lycopene and β-carotene, which are associated with important health benefits. However, tomato production is frequently limited by a wide range of bacterial, oomycete, fungal, and viral pathogens that cause significant yield loss and economic damage. Global tomato production continues to expand to meet the demand from both fresh markets and processing industries (WPTC; Kaul et al., 2025), increasing the need for durable and sustainable disease resistance strategies.

Conventional breeding approaches for disease resistance are often slow and constrained by limited genetic diversity and linkage drags. CRISPR/Cas9 (Clustered Regularly Interspaced Short Palindromic Repeats/Cas9) genome editing is a precise and efficient alternative for introducing targeted genetic modifications (Erdoğan et al., 2023; Tiwari et al., 2023). This technology enables the rapid functional analysis of genes and the development of improved cultivars with desirable agronomic traits. CRISPR/Cas9 has been widely applied in tomatoes to modify traits, including disease resistance, demonstrating its versatility as a tool for crop improvement (Thomazella et al., 2021; Nekrasov et al., 2017; Tashkandi et al., 2018; Ortigosa et al., 2019; Ortega et al., 2024; Sandhya et al., 2025).

Here, we describe a detailed and reproducible protocol for generating disease-resistant tomato plants through targeted editing of *susceptibility* (*S*) genes using the CRISPR/Cas9 system. The protocol integrates guide RNA (gRNA) design and off-target prediction using CRISPOR, Golden Gate cloning, *Agrobacterium*-mediated transformation, optimized tissue culture-based regeneration, and standardized molecular validation of edited plants. By combining optimized regeneration procedures with quantitative benchmarks and sequencing-based mutation analysis, this workflow provides an integrated and experimentally validated pipeline for producing targeted gene knockouts. This protocol is designed to support both functional genomics research and the practical implementation of genome-editing strategies for developing improved tomato varieties.

## 2 Materials and methods

### 2.1 Materials

#### 2.1.1 Laboratory supplies and consumables

Pipette sets and sterile tips, 0.5- and 1.5-mL microcentrifuge tubes, PCR tubes, disposable plate spreaders and inoculation loops, tube racks, sterile distilled water, ice buckets, personal protective equipment (gloves and masks), and standard laboratory disinfectants were used throughout the experiments.

#### 2.1.2 Reagents and media

LB broth and LB agar, antibiotics (including spectinomycin, ampicillin, chloramphenicol, kanamycin, rifampicin, and gentamicin), Murashige and Skoog (MS) medium, plant growth regulators (IAA, BAP, zeatin, and IBA), timentin, agar (0.7%), SOC medium, and acetosyringone (ACS) were used.

#### 2.1.3 Molecular biology reagents

BpiI and BsaI restriction enzymes, T4 DNA ligase, plasmid DNA isolation kits, PCR reagents and primers, and Sanger DNA sequencing services were used for molecular cloning and validation of the constructs.

#### 2.1.4 Biological materials

Tomato (*S. lycopersicum*) cv. “Ailsa Craig” seeds, CRISPR/Cas9 vectors (pDGE5, pDGE8, and pDGE1), *Agrobacterium tumefaciens* strain GV3101, electrocompetent *Escherichia coli* DH10b cells, and electroporation cuvettes were used.

#### 2.1.5 Equipment

A thermal cycler (PCR machine), BioRad MicroPulser electroporator, centrifuge, and controlled-environment growth chambers were used.

### 2.2 gRNA design and vector construction

**Figure 1:**
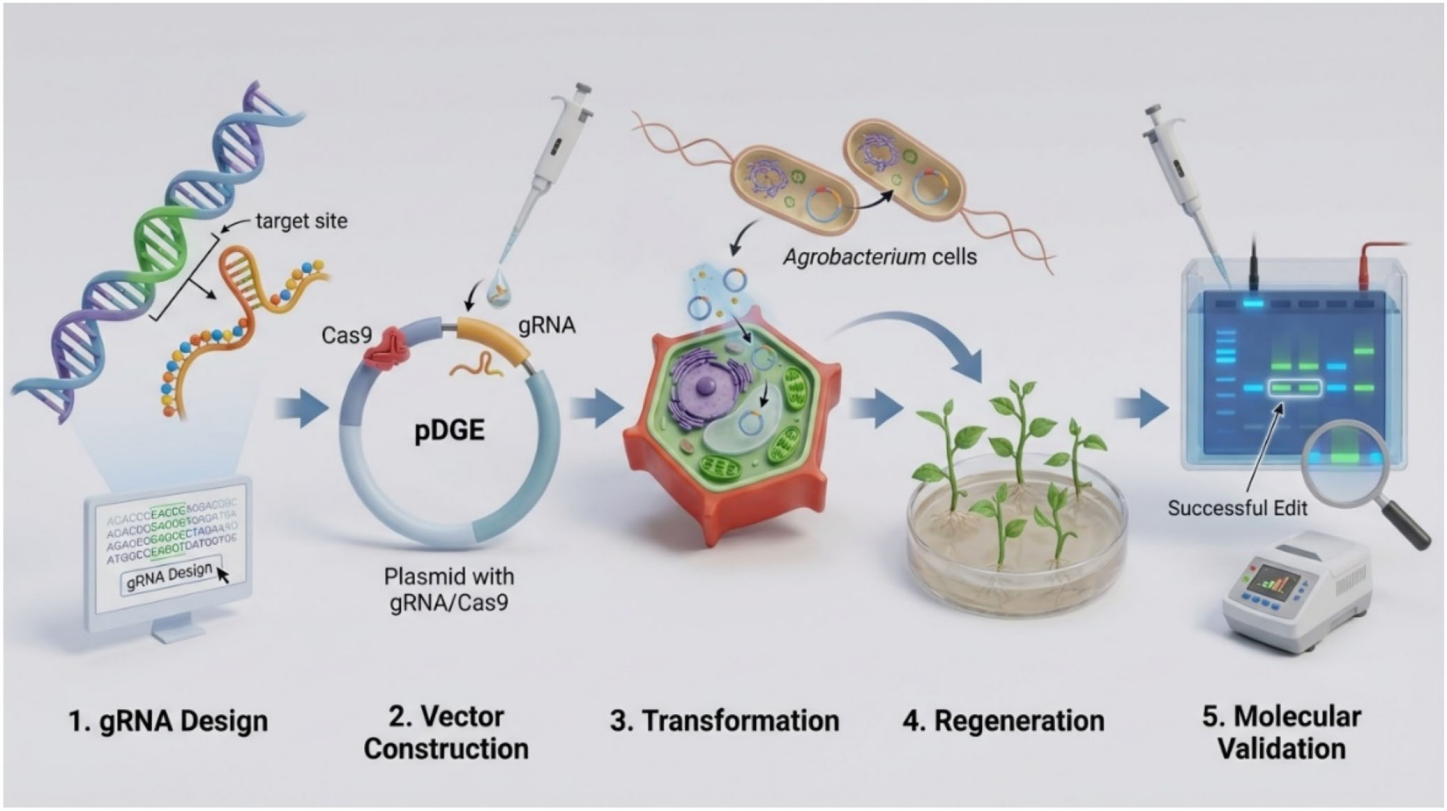
CRISPR/Cas9 workflow in tomato. Schematic overview of the integrated genome editing workflow, including gRNA design using CRISPOR, Golden Gate vector assembly, *Agrobacterium*-mediated transformation, plant regeneration, and molecular validation of edited plants.

gRNAs targeting the tomato susceptibility gene *MLO1* were identified using the NCBI database (O’Leary et al., 2024). gRNAs were designed using the CRISPOR web tool (Concordet and Haeussler, 2018) based on the presence of PAM, GC content, and predicted specificity scores. Potential off-target sites were evaluated *in silico* using the tomato reference genome. The overall workflow used for CRISPR/Cas9-mediated genome editing and plant regeneration is illustrated in **Figure 1**.

gRNA sequences:

gRNA1 Forward: ATTGGGAGGTACCACGCAATGGTG.
gRNA1 Reverse: AAACCACCATTGCGTGGTACCTCC.
gRNA2 Forward: ATTGCCATGGTTAGCCTTATGGCT.
gRNA2 Reverse: AAACAGCCATAAGGCTAACCATGG.

#### 2.2.1 Oligonucleotide hybridization

Lyophilized oligonucleotides were resuspended to 100 µM in sterile water and then diluted to 10 µM. Equal volumes (5 µL each) of forward and reverse oligonucleotides were mixed with 40 µL of sterile water to obtain a 5 µM hybridization solution. The mixture was denatured at 98 °C for 5 min and allowed to cool to room temperature (RT). A 1:100 dilution (50 fmol/µL) was prepared for cloning and stored at −20°C.

#### 2.2.2 Golden Gate cloning

**Figure 2:**
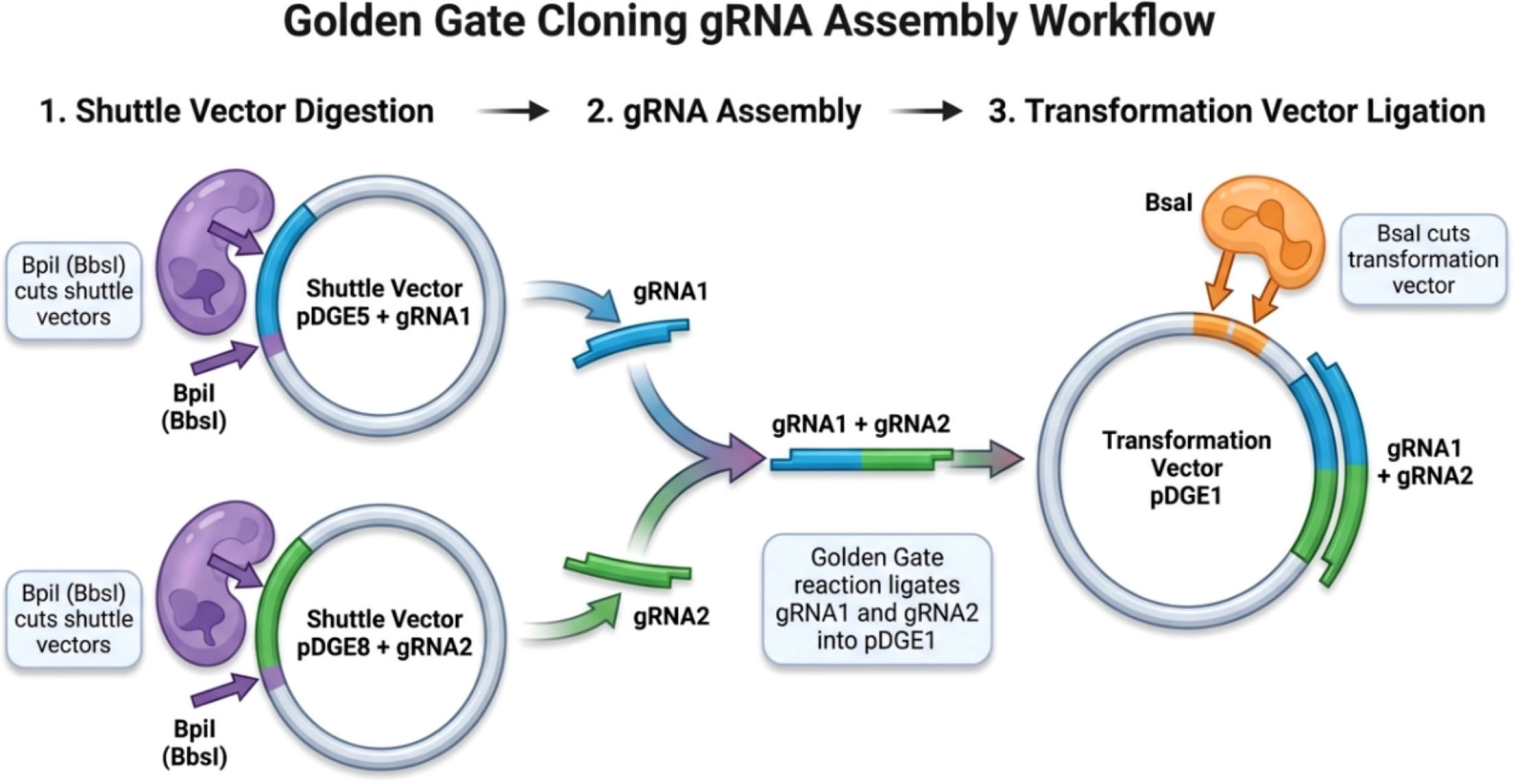
Golden Gate cloning strategy. Diagram illustrating the assembly of gRNA constructs using pDGE5 and pDGE8 shuttle vectors, followed by integration into the final pDGE1 binary vector (Ordon et al., 2017; Stuttmann et al., 2021).

**Figure 3:**
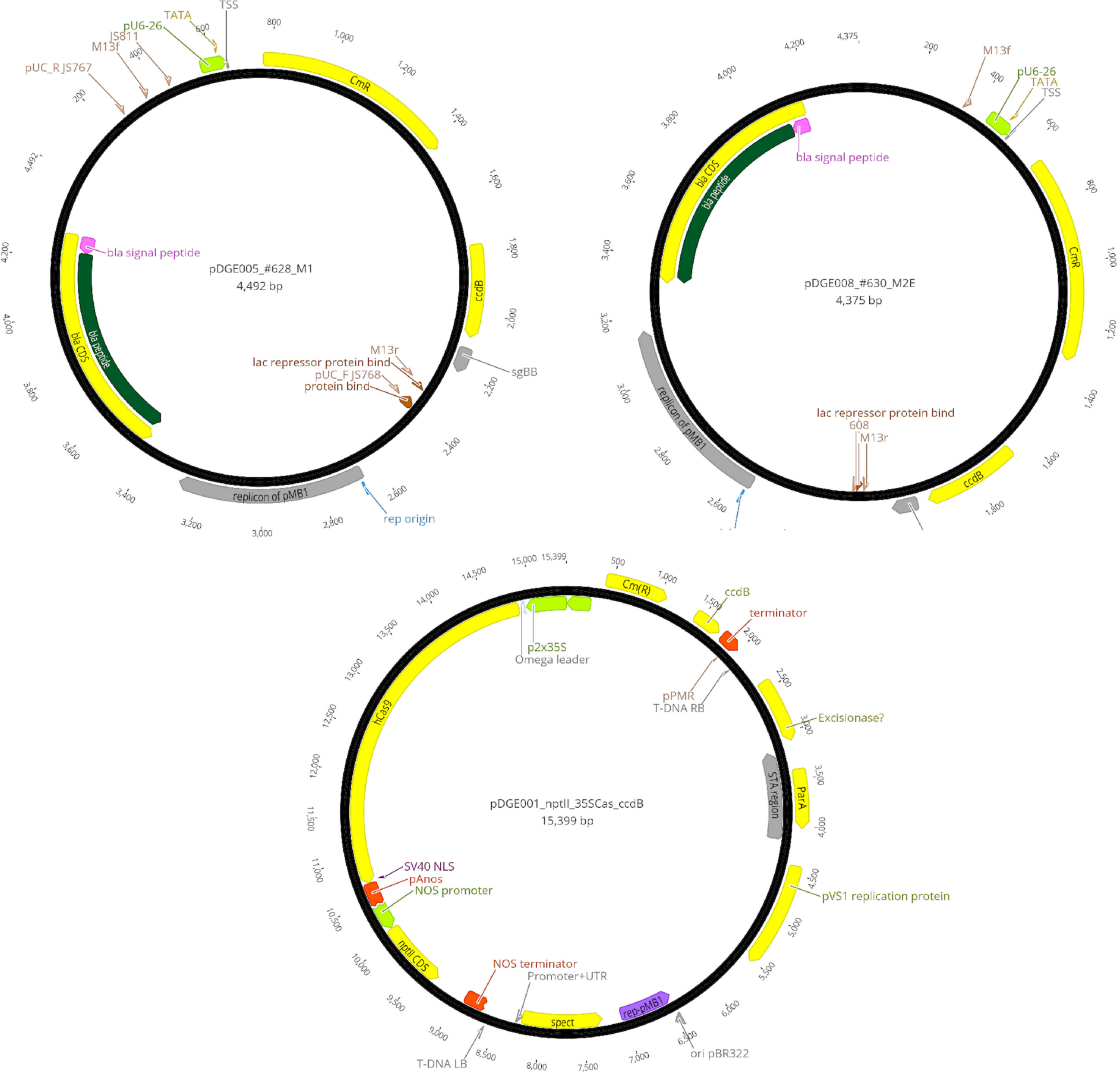
CRISPR/Cas9 vector maps. **(A)** pDGE5 shuttle vector; **(B)** pDGE8 shuttle vector; **(C)** pDGE1 final transformation binary vector (Ordon et al., 2017; Stuttmann et al., 2021).

gRNA1 and gRNA2 were first cloned into shuttle vectors pDGE5 and pDGE8, respectively, and subsequently assembled into the final binary vector pDGE1 using Golden Gate cloning (Ordon et al., 2017; Stuttmann et al., 2021). The Golden Gate cloning strategy used for gRNA assembly into the transformation vector is illustrated in **Figure 2**, and the plasmid constructs used for genome editing are shown in **Figure 3**.

Cut–ligation reaction for shuttle vectors (10 µL total volume):

4 µL of dH_2_O.
3 µL of pDGE5 or pDGE8 (20 fmol ≈ 200 ng).
1 µL of hybridized oligonucleotides.
1 µL of 10× ligation buffer.
0.5 µL of BpiI (10U/µL).
0.5 µL of T4 DNA ligase (10U/µL).

Final vector assembly (20 µL total volume):

11.5 µL of dH_2_O.
3.5 µL of pDGE1 (200 ng).
0.5 µL of pDGE5-gRNA1 (20 fmol ≈ 220 ng).
0.5 µL of pDGE8-gRNA2 (20 fmol ≈ 220 ng).
2 µL of 10× ligation buffer.
1 µL of BsaI (10U/µL).
1 µL of T4 DNA ligase (10U/µL).

The thermocycling conditions were as follows: 37 °C for 2 min and 16 °C for 5 min (10–30 cycles), followed by 50 °C for 10 min and 80 °C for 10 min. The reaction products were purified using spin columns and stored at 4 °C.

#### 2.2.3 Transformation into *E. coli*

Electrocompetent *E. coli* DH10b cells (15 µL) were mixed with 3 µL of purified ligation product and electroporated at 2.5 kV (cuvette gap: 0.1 cm). Cells were recovered in 250 µL of SOC medium at 37°C for 1 h with shaking and plated on LB agar containing spectinomycin. Single colonies were cultured overnight, and plasmid DNA was isolated using a miniprep kit.

### 2.3 *Agrobacterium*-mediated transformation

Purified plasmids were introduced into electrocompetent *A. tumefaciens* GV3101 by electroporation. Cells were recovered in SOC medium at 28 °C for 1 h and plated on LB agar containing spectinomycin, gentamicin, and rifampicin. The colonies were cultured in selective LB broth and stored as glycerol stocks.

### 2.4 Tomato regeneration and selection

**Figure 4:**
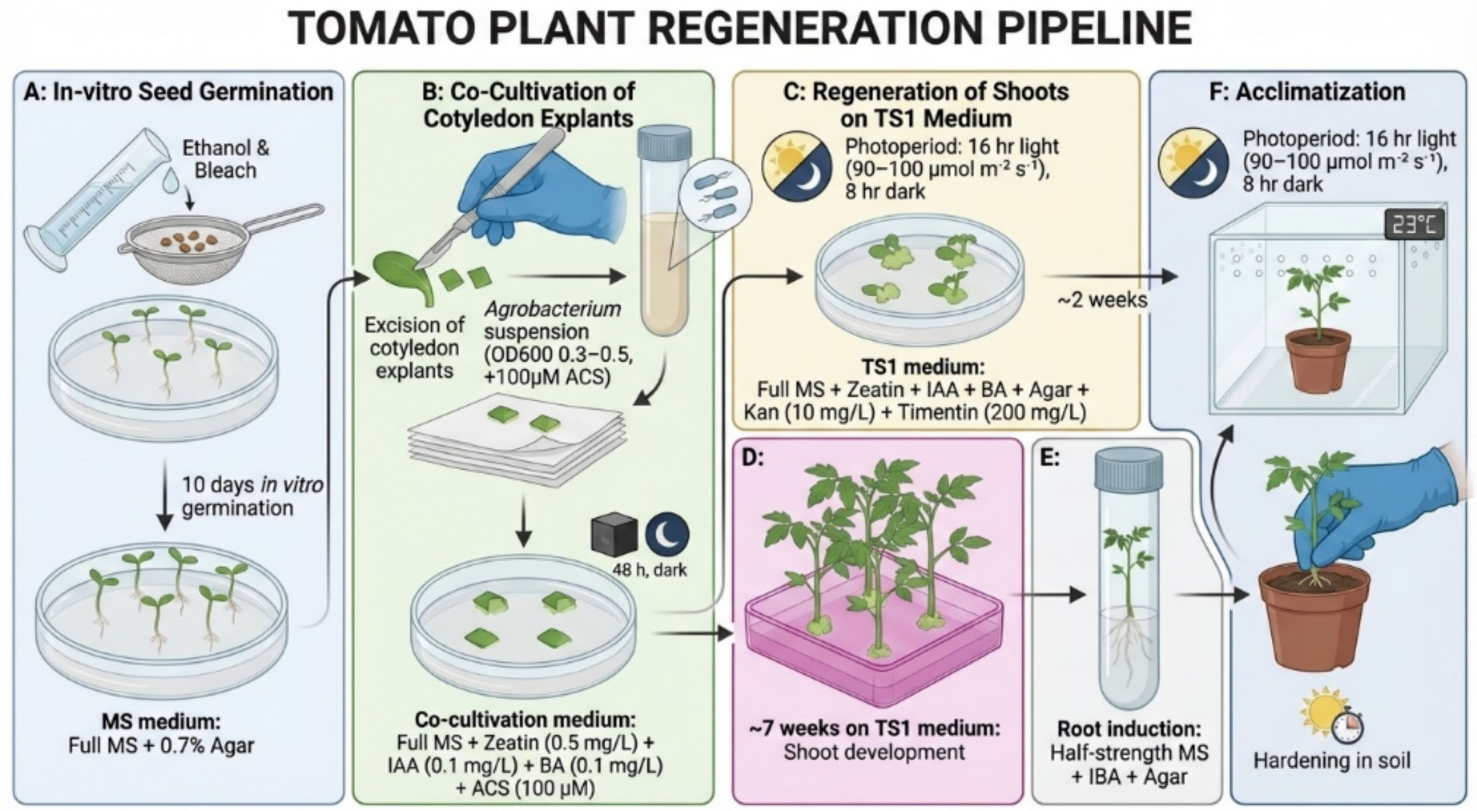
Tomato regeneration pipeline. Sequential stages of tomato transformation and regeneration following *Agrobacterium*-mediated gene transfer. **(A)** In vitro seed germination. **(B)** Co-cultivation of cotyledon explants with Agrobacterium. **(C)** Shoot regeneration on TS1 medium. **(D)** Shoot development. **(E)** Root induction. **(F)** Acclimatization and hardening of regenerated plants in soil.

The tomato regeneration and transformation pipeline used in this study is summarized in **Figure 4**. Sterilized tomato seeds were germinated *in vitro* on full-strength MS medium with 0.7% agar for 10 days. Cotyledon explants were co-cultivated with *Agrobacterium* suspension (OD_600_ 0.3–0.5) supplemented with 100 µM ACS for 5–10 min and incubated on co-cultivation medium in the dark for 48 h.

The explants were transferred to shoot induction medium (TS1), which consisted of full-strength MS supplemented with zeatin (0.5 mg/L), IAA (0.1 mg/L), BAP (0.1 mg/L), kanamycin (10 mg/L), and timentin (200 mg/L). The cultures were maintained under a 16-h light/8-h dark photoperiod. Shoots emerged approximately 2 weeks after co-cultivation and were maintained on TS1 medium for approximately 7 weeks.

**Figure 5:**
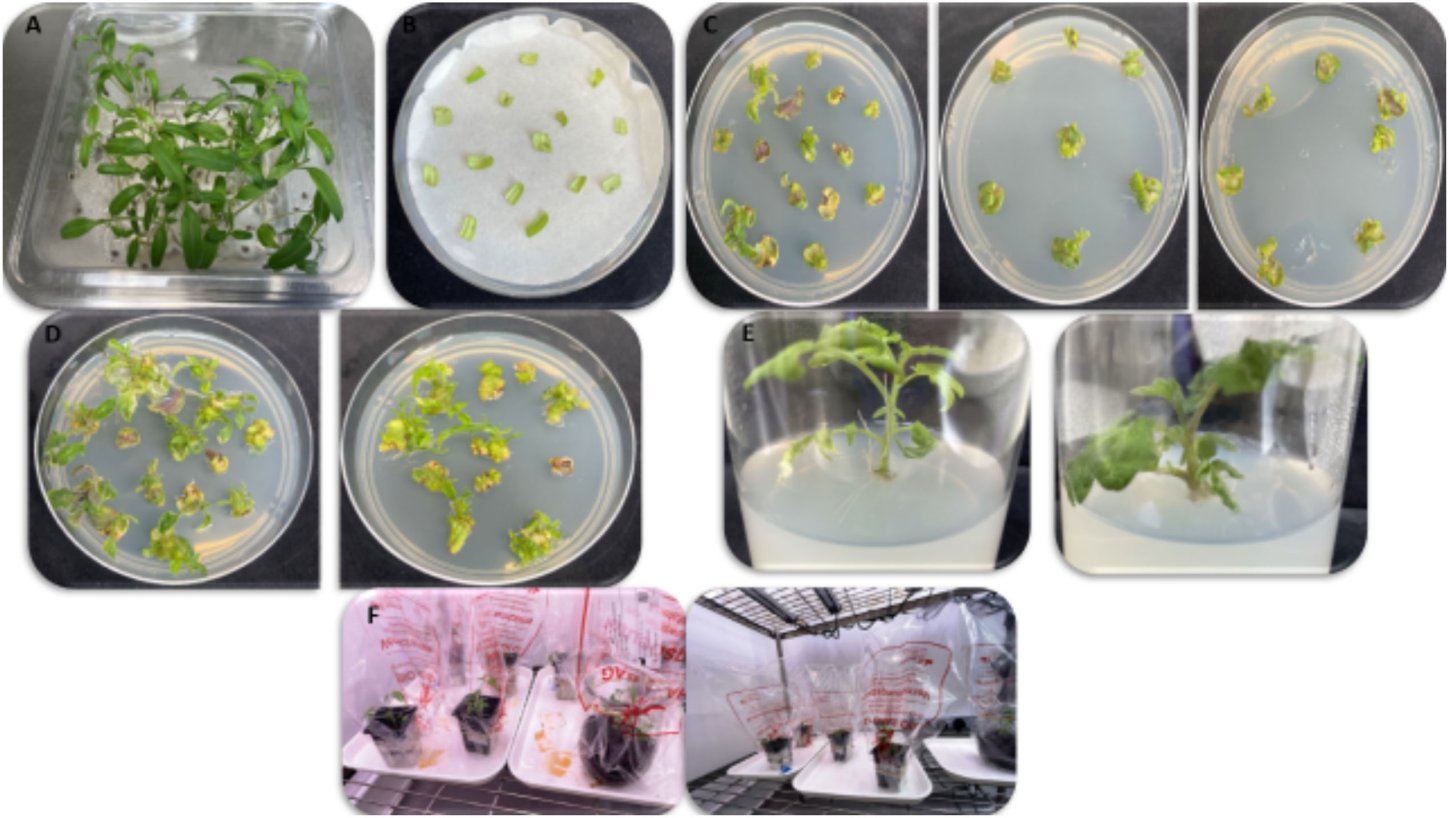
Stage-wise regeneration of tomato plants. **(A)** *In vitro* seed germination; **(B)** co-cultivation of cotyledon explants; **(C)** callus formation and shoot regeneration; **(D)** shoot development on TS1 medium; **(E)** root induction; **(F)** acclimatization of regenerated plantlets.

Regenerated shoots were transferred to half-strength MS rooting medium supplemented with IBA and subsequently acclimatized in soil under controlled growth chamber conditions (23 °C, long-day photoperiod) (**Figure 5**).

Regeneration, transformation, and editing efficiencies were calculated based on the number of co-cultivated explants and confirmed edited plants.

### 2.5 gRNA off-target analysis

Off-target predictions were performed using CRISPOR. Candidate sites with ≤3 mismatches and canonical PAM sequences were evaluated. The specificity scores and predicted off-target counts are summarized in **Table 1**.

**Table 1:** Predicted off-target specificity metrics and off-target counts for guide RNAs targeting MLO1 locus.

Position/Strand	Guide sequence + PAM	mitSpecScore	cfdSpecScore	Off-targets count
329rev	GGAGGTACCACGCAATGGTGTGG	100	100	0
111rev	CCATGGTTAGCCTTATGGCTAGG	95	98	12

### 2.6 Molecular screening and genome editing validation

Genomic DNA was extracted from the regenerated plants using a modified Shorty DNA extraction protocol (Edwards et al., 1991). DNA quality was assessed using agarose gel electrophoresis.

Genome editing in kanamycin-resistant T0 plants was validated by PCR amplification of the target locus, followed by Sanger sequencing. Sequence traces were analyzed using DSDecodeM and TIDE to determine the indel patterns and mutation frequencies. Predicted protein consequences were evaluated using the ExPASy Translate tool, which revealed frameshift mutations and premature stop codons in the edited lines.

## 3 Results

### 3.1 PCR confirmation of CRISPR/Cas9 integration

**Figure 6:**
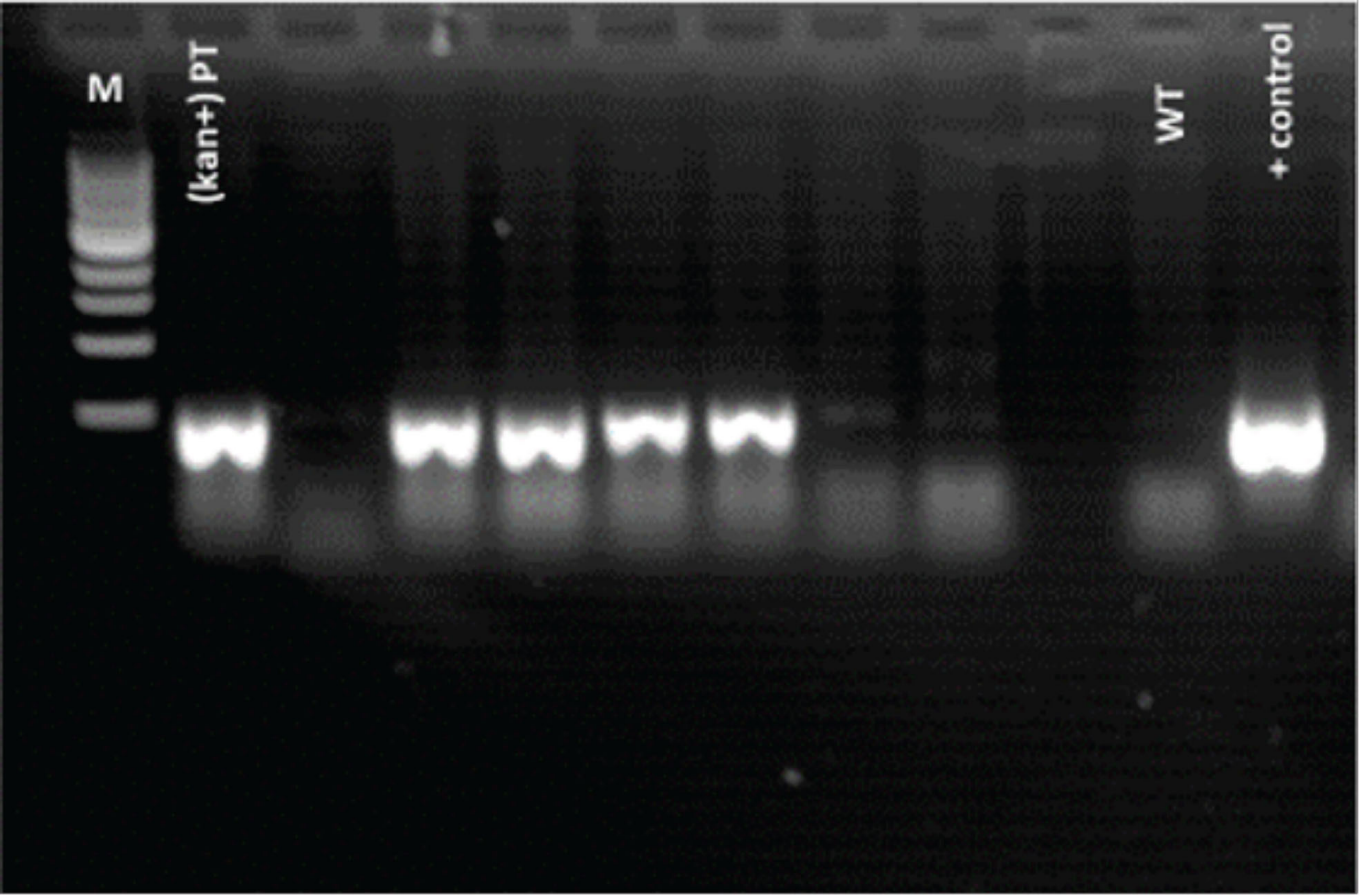
PCR confirmation of putative transformants. Examples of PCR amplification using kanamycin-specific primers to confirm construct integration. M, DNA marker; kan^+^ PT, kanamycin-positive putative transformants; WT, wild-type Ailsa Craig.

The integration of the CRISPR/Cas9 construct into the regenerated tomato plants was verified using PCR with kanamycin-specific primers [Forward: CTTCCCGCTTCAGTGACAAC, Reverse: TTGGGTGGAGAGGCTATTCG]. Amplification of the expected fragment was observed in kanamycin-positive transformants (kan^+^ PT), whereas wild-type (WT) plants showed no band (**Figure 6**). These results confirmed the successful T-DNA integration in the selected T_0_ plants.

PCR amplification was performed in 25-µL reaction volumes. Each reaction contained 12.5 µL of Red Mix, 0.5 µL of forward primer, 0.5 µL of reverse primer, and nuclease-free dH_2_O adjusted to volume, with 1 µL of template DNA added per reaction. Reactions were prepared as a master mix for 10 samples to minimize the pipetting variability. PCR was carried out under the following cycling conditions: initial denaturation at 95°C for 1 min; 30 cycles of denaturation at 95°C for 15 s, annealing at 60°C for 15 s, and extension at 72°C for 10s; followed by a final extension at 72°C for 5 min. Amplified products were stored at 4°C until further analysis.

### 3.2 Sequence-based detection of indels

To detect the targeted mutations, genomic regions flanking the gRNA target sites were amplified using the following primers:

Forward: ATGATCAGTGGAGGCATGCT.
Reverse: GGAGTTGGGTAAGGAGTTGGA.

**Figure 7:**
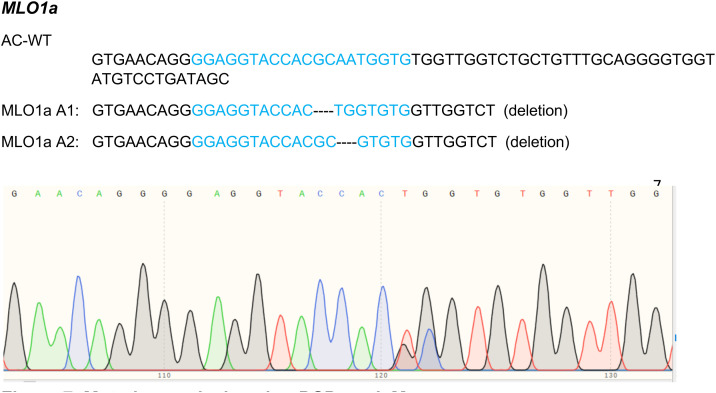
Mutation analysis using DSDecodeM. Representative deletion patterns in kanamycin-positive plants. AC-WT: wild-type sequence; A1 and A2: edited alleles. Blue sequences indicate the gRNA target sites. Deletions are represented by dashed lines. Biallelic mutations were observed in several lines, indicating effective genome editing at the target locus.

Targeted regions flanking the gRNA sequences were PCR-amplified and Sanger-sequenced. Analysis with DSDecodeM revealed various indel patterns in the kanamycin-positive plants. Representative alleles of *MLO1* are shown in **Figure 7**.

### 3.3 TIDE analysis of indel frequency

**Figure 8:**
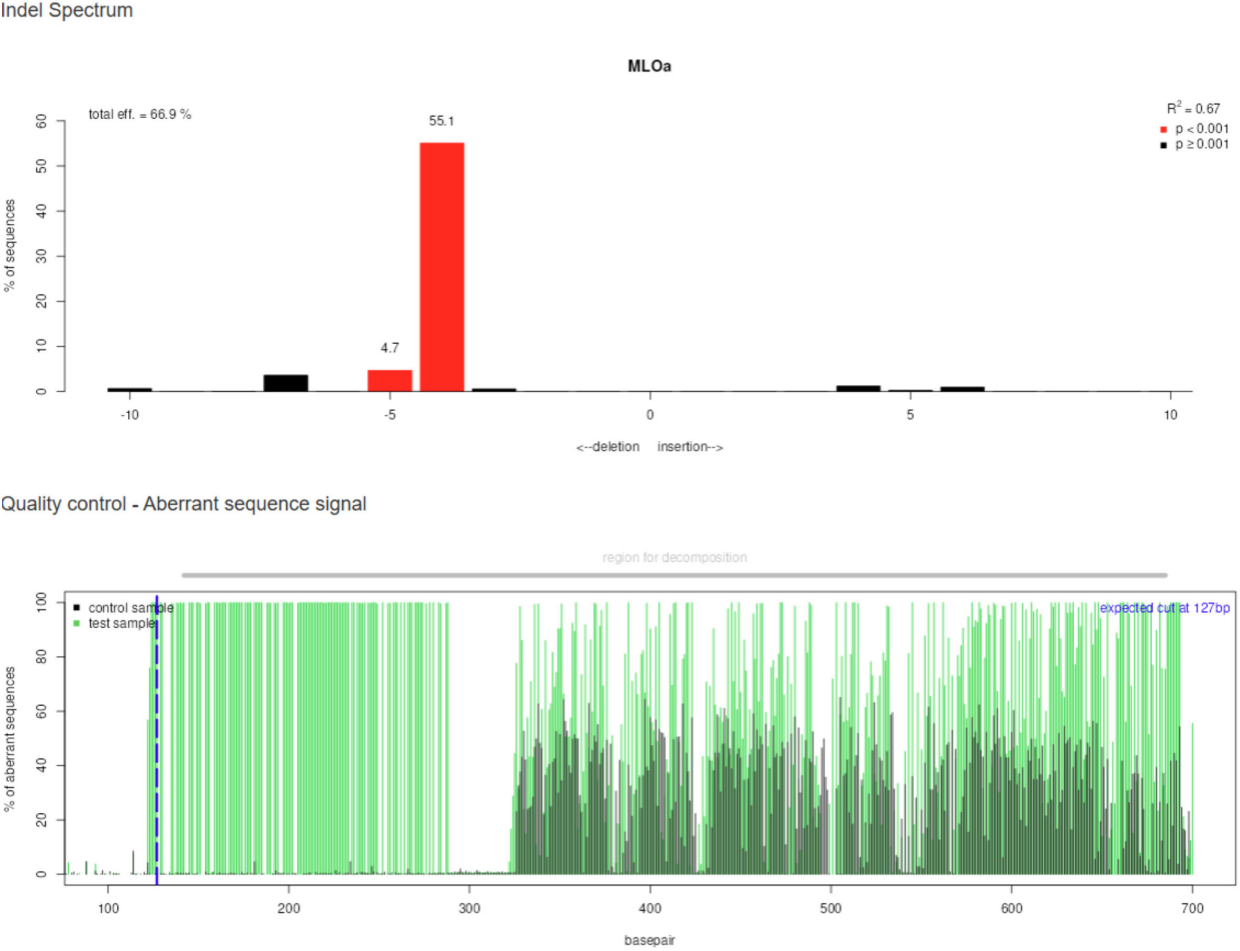
TIDE analysis of genome-edited plants. Decomposition of indel frequencies in T_0_ plants, showing mutation efficiency and distribution of insertion/deletion events.

Indel frequencies were further quantified using TIDE, which decomposes sequencing traces to determine insertion/deletion distributions and mutation efficiencies. TIDE analysis (**Figure 8**) indicated editing efficiencies ranging from 40% to 70% in T_0_ plants, consistent with PCR and DSDecodeM data. These data demonstrate the reproducibility of the workflow across multiple transformants.

### 3.4 Predicted protein consequences

**Figure 9:**
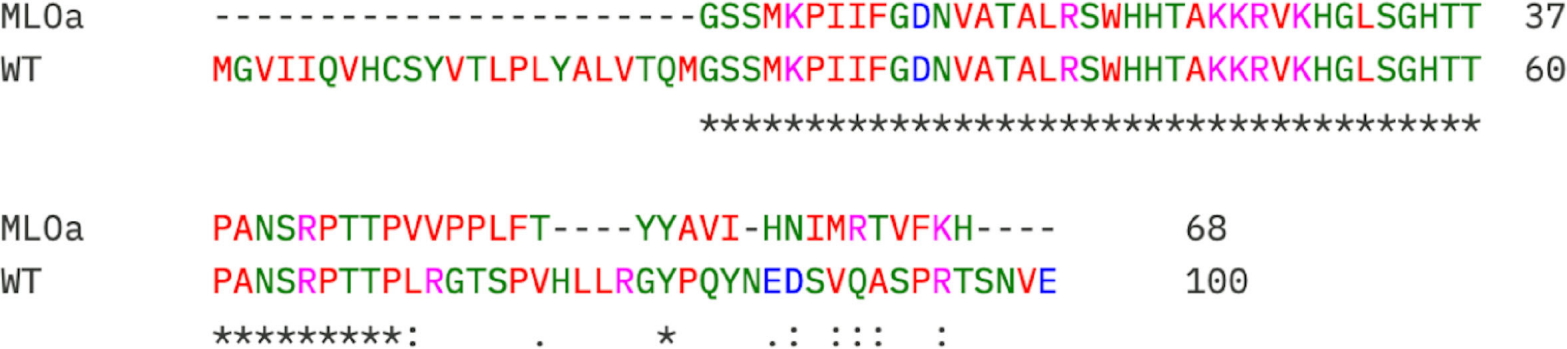
Amino acid sequence alignment of MLOa and wild-type (WT) proteins. Identical residues are indicated by asterisks (*), conserved substitutions are denoted by colons (): and semi-conserved substitutions are represented by periods (.). The gaps introduced to maximize alignment are shown as dashes (-). Residues are colored according to amino acid properties: hydrophobic residues in green, polar uncharged residues in purple, positively charged residues in blue, and negatively charged residues in red.

Translation of the edited alleles using the ExPASy Translate tool revealed frameshift mutations that introduced premature stop codons (**Figure 9**). These mutations were predicted to truncate the encoded proteins, consistent with the generation of loss-of-function alleles in *MLO1*.

### 3.5 Transformation, regeneration, and editing efficiency

**Table 2** summarizes the number of co-cultivated explants, regeneration, transformation efficiency based on Kan diagnostic PCR, and editing efficiency of the transformants.

**Table 2:** Regeneration, transformation, and editing efficiencies of the *MLO1* gene-editing construct.

Gene construct	No. of co-cultivated explants	Regeneration efficiency (%)	Transformation efficiency (%)	Editing efficiency (%)
*MLO1*	75	62.70	37.33	50

**Table 1** lists the gRNA specificity scores and predicted off-target counts from the CRISPOR analysis. No high-probability off-target sites were detected for gRNA1, whereas gRNA2 had minimal predicted off-targets (≤12 with low specificity risk), supporting the high precision of the designed gRNAs, ranked by default from highest to lowest specificity score (Hsu et al., 2013).

## 4 Discussion

### 4.1 Methodological advancement

This protocol comprises a facile process for groups to establish gene-editing experiments in tomato plants. It integrates optimized regeneration conditions, Golden Gate cloning, molecular validation (PCR and sequencing), and bioinformatic assessment (DSDecodeM, TIDE, and ExPASy) into a reproducible pipeline for CRISPR/Cas9-mediated genome editing in tomato. Quantitative benchmarks for transformation, regeneration, and editing efficiency allow researchers to replicate and adapt this workflow.

### 4.2 Reproducibility and validation

The combination of PCR confirmation, sequencing-based indel detection, and TIDE analysis provides the user with a robust, multilayered validation strategy. Predicted protein-level consequences ensure that the observed mutations are functionally relevant, enabling reliable knockout generation.

### 4.3 Expected outcomes

- Shoot regeneration occurs within 2–3 weeks post-transformation, and rooted plantlets are ready for acclimatization in 6–8 weeks.
- Editing efficiencies typically range from 40% to 70%, depending on the gRNA design and tomato genotype.
- Homozygous or biallelic T_0_ plants displayed frameshift mutations and premature stop codons, consistent with loss of function.
- Transgene-free edited lines can be obtained in subsequent generations by segregation.
- Phenotypic assays under pathogen challenge (powdery mildew, *Oidium neolycopersici*) are expected to demonstrate enhanced disease resistance in knockout lines compared to that in the WT.

### 4.4 Best practices

- Select gRNAs with high predicted efficiency and minimal off-target effects.
- Maintain strict aseptic technique during tissue culture.
- Always include WT and empty vector controls at all stages of the experiment.
- Whole-genome sequencing may be considered for comprehensive off-target analyses if end objectives are required.

### 4.5 Applicability

This workflow is broadly adaptable to other tomato cultivars or related Solanaceae crops with minor modifications. This protocol facilitates functional genomic studies and accelerates the generation of disease-resistant tomato varieties (**Table 3**). #Common technical issues encountered during the tomato transformation and regeneration workflow, along with suggested troubleshooting strategies, are summarized in **Table 3**.

**Table 3:** Troubleshooting.

Step	Problem	Possible cause	Solution
gRNA cloning	Low ligation efficiency	Incomplete digestion or inefficient ligase	Verify enzyme activity, increase ligation time or temperature cycles
*E. coli* transformation	No colonies after electroporation	Poor competency or electrical settings	Use freshly prepared competent cells, ensure correct electroporation parameters (or provide)?
*Agrobacterium* transformation	No growth on selective plates	Antibiotic concentrations too high or plasmid lost	Confirm antibiotic stock concentrations, recheck plasmid integrity (sequence)
Shoot regeneration	Poor shoot induction	Suboptimal PGR concentrations or dark period	Optimize Zeatin/IAA/BA ratios, ensure 48 h dark co-cultivation
High contamination	Bacterial/fungal contamination in culture	Poor aseptic technique or non-sterile seeds	Use 70% ethanol and bleach sterilization for seeds, work in laminar flow cabinet
Low editing efficiency	No mutations detected	gRNA not effective or low Cas9 activity	Redesign gRNA targeting exonic conserved regions, verify expression with RT-PCR
Chimerism in T_0_ plants	Mosaic editing due to late Cas9 activity	Common in first-generation CRISPR plants	Screen T_1_ generation to identify stable lines, ideally vector free lines
No root formation	Shoot death on rooting medium	Hormone imbalance or bacterial overgrowth	Adjust IBA concentration, use fresh media, check for latent *Agrobacterium*

## Data Availability

The original contributions presented in the study are included in the article/supplementary material. Further inquiries can be directed to the corresponding author.
